# Targeting Tumour-Associated Fibroblasts in Cancers

**DOI:** 10.3389/fonc.2022.908156

**Published:** 2022-06-22

**Authors:** Kairav Shah, Sanchari Basu Mallik, Praveer Gupta, Abishek Iyer

**Affiliations:** Alembic Discovery & Innovation, Alembic Pharmaceuticals, Hyderabad, India

**Keywords:** cancer-associated fibroblasts, tumour microenvironment, inflammation, cancer, immunotherapy

## Abstract

Tumours develop within complex tissue environments consisting of aberrant oncogenic cancer cells, diverse innate and adaptive immune cells, along with structural stromal cells, extracellular matrix and vascular networks, and many other cellular and non-cellular soluble constituents. Understanding the heterogeneity and the complex interplay between these cells remains a key barrier in treating tumours and cancers. The immune status of the pre-tumour and tumour milieu can dictate if the tumour microenvironment (TME) supports either a pro-malignancy or an anti-malignancy phenotype. Identification of the factors and cell types that regulate the dysfunction of the TME is crucial in order to understand and modulate the immune status of tumours. Among these cell types, tumour-associated fibroblasts are emerging as a major component of the TME that is often correlated with poor prognosis and therapy resistance, including immunotherapies. Thus, a deeper understanding of the complex roles of tumour-associated fibroblasts in regulating tumour immunity and cancer therapy could provide new insight into targeting the TME in various human cancers. In this review, we summarize recent studies investigating the role of immune and key stromal cells in regulating the immune status of the TME and discuss the therapeutic potential of targeting stromal cells, especially tumour-associated fibroblasts, within the TME as an adjuvant therapy to sensitize immunosuppressive tumours and prevent cancer progression, chemo-resistance and metastasis.

## 1 Introduction

**Figure 1:**
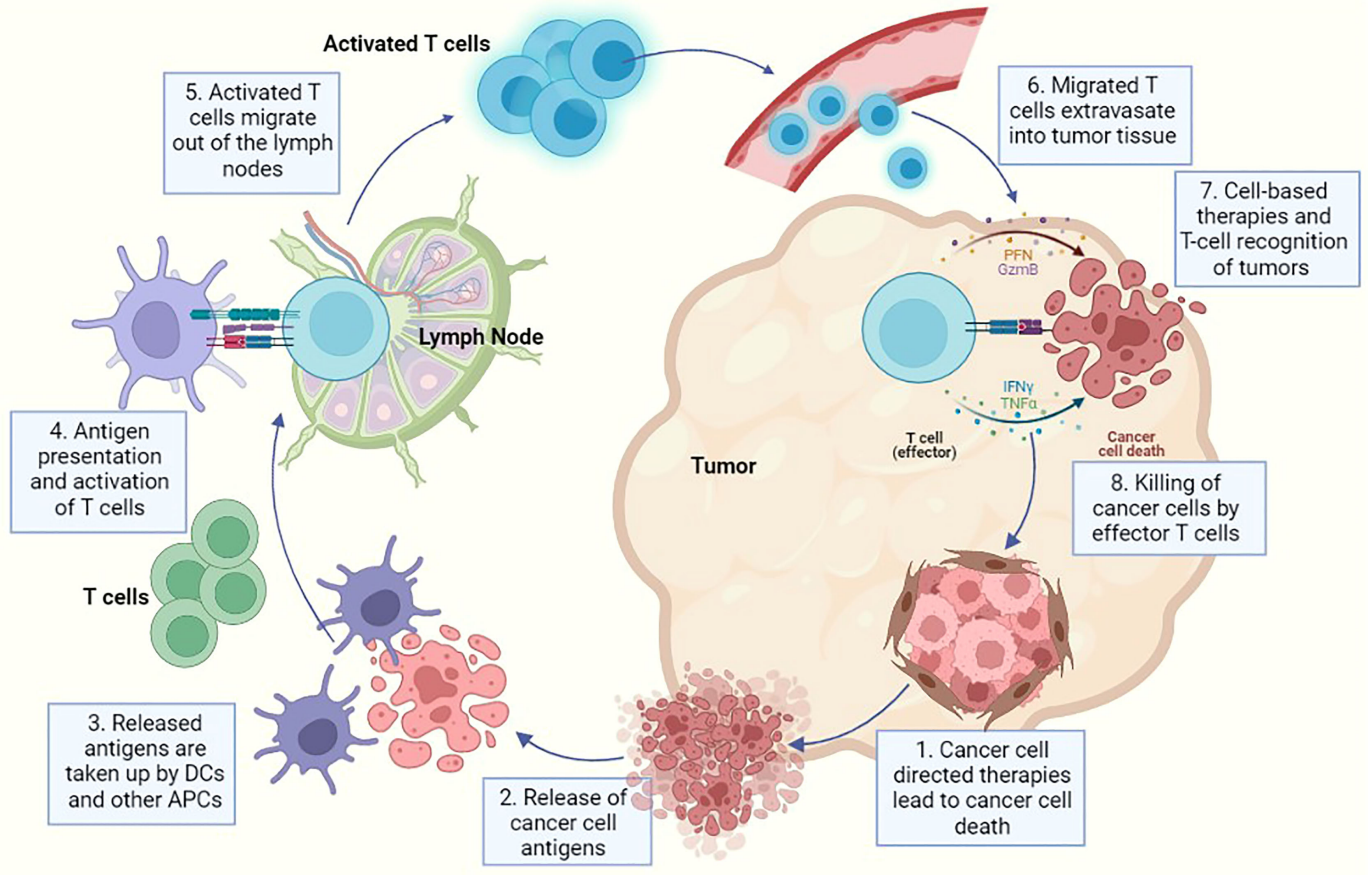
A representative diagram for cancer progression and associated immune reaction where each step indicates a potential method of therapeutic intervention using either cancer-cell directed therapies or immune cell directed therapies. In this image - 1. Indicates killing of tumour cells using cancer-cell directed therapies like chemotherapy, radiation etc., 2. Represents the release of cancer cell specific antigens after cancer cell death has been induced, 3. Represents the cancer antigen presentation to dendritic cells and antigen presenting cells, 4. Indicates priming of APCs and T cell activation, 5. Demonstrates the migration of activated T cells from the lymph node into the circulation, 6. Represents the migration of T cells into tumour tissue *via* extravasation through endothelial cells in blood vessels, 7. Demonstrates the recognition of cancer cells by effector T cells and also represents the point of action of multiple cell based therapies and **(8)**. Finally represents the killing of cancer cells after recognition by effector T cells. Multiple immunotherapies have been developed capitalizing on this particular step e.g. checkpoint inhibitors, metabolic reprogramming therapies etc.

The tumour and its microenvironment (TME) not just consists of aberrant oncogenic cancer cells but are additionally composed of recruited and resident host cells such as various immune cells and other structural stromal cells (1). The tumour stroma contains components of extracellular matrix (ECM) and a host of different cell types such as immune cells, fibroblasts and vascular endothelial cells. For a long time, a cancer cell centric view of tumours led us to believe that mutations in oncogenes and tumour suppressor genes were sufficient to determine tumour survival, growth and progression. However, the current paradigm is that cancer cell derived signals delivered to immune and stromal cells reprogram the phenotype of these cells and determine, to a large extent, the survival and proliferation of oncogenic cancer cells. The exact phenotype and polarisation state of these stromal and immune cells will determine the immune status of the TME and further dictate tumour initiation, progression and metastasis (2). This change in paradigm from a cancer cell centric view to a TME centric view has opened new avenues for cancer therapy. Indeed, even conventional, tumour targeted therapies such as cytotoxic chemotherapies derive significant clinical benefit from their ability to stimulate anticancer immunity upon inducing cancer cell death, by increasing the tumour’s antigenicity and adjuvanticity (**Figure 1**). Further, the understanding that the TME is capable of reprograming and normalising tumour cells has led to development of novel approaches such as re-educating and reprograming immune and other stromal cells to prevent tumour progression and induce effective cancer remission (3).

Apart from malignant tumour cells, various types of resident sentinel and infiltrated innate and adaptive immune cells, and stromal cells such as vascular endothelial cells, tumour-associated fibroblasts (TAFs), together with extracellular matrix (ECM) as well as multiple extracellular soluble factors such as cytokines, proteases, chemotactic factor, growth factors and others, are now recognized as part of the TME (4). The recent evolution of single cell technologies has provided significant insights into the heterogenous nature of the TME. Studies focused on single cell RNA sequencing (scRNA-seq) have yielded data on the diversity of cellular phenotypes in the TME, revealing a wealth of new targets as well as unknown cell types that can potentially play a pivotal role in cancer progression (5). Developed tumours that have a pro-inflammatory milieu and increased ratio of tumour fighting effector cells compared to tumour promoting immunosuppressive cells are often referred to as ‘Hot’ tumours. On the other hand, when the balance tilts towards tumour promoting immunosuppressive cells, these tumours are referred to as ‘Cold’ tumours (4). Understanding and identifying the key factors and cell types that regulate cold to hot transition of the TME, and vice versa, is an intense area of investigation. Key innate immune cells such as macrophages, dendritic cells and neutrophils have been prime suspects, as these cells are notorious for their functional plasticity and have been found in both hot and cold TMEs. Among these cells, tumour macrophages are the most studied to date and the key mechanisms and approaches to re-educate these cells in the TME have been extensively reviewed elsewhere (6). However, apart from macrophages and other immune cells, stromal cells represent a large, often under-appreciated cell population present in the TME that could be re-educated to prevent tumour progression and induce effective cancer remission. Activated fibroblasts trained by cancer cells, called tumour associated fibroblasts (TAFs or CAFs), consist of a large proportion of this stromal component (7). TAFs have largely been characterized as master controllers of matrix remodelling in tumours as they are the major producers of collagen, other matrix proteins and enzymes. Emerging evidence suggests that cells of the fibroblast lineage play a central role in tumour-related inflammation by engaging in complex interactions with cancer cells, as well as other stromal and tumour resident and infiltrating immune cells. These cells promote cancer cell proliferation and survival, angiogenesis and suppress anti-tumour T-cell responses. Several factors, including hypoxia, chemokines, cytokines and metabolic products of cancer cells (for example, lactic acid) are involved in TAF activation and functional polarization. Recent evidence indicates that TAFs also bear significant responsibility in influencing tumour metabolism. They may also play an important role in the hot to cold tumour immune phenotype transition in the TME, that represents one of the biggest challenges in cancer immunotherapy (8). In this review, we will describe known fibroblast biology and summarise the multifaceted roles that TAFs play in cancer progression and in sculpting an immunosuppressive tumour-microenvironment. We will also evaluate the rationale for targeting TAFs in combination with current immunotherapies, and explore current strategies employed in the field to re-educate and reprogram TAFs.

## 2 Tumour-Associated Fibroblasts

### 2.1. Fibroblasts in the Tumour Microenvironment

**Figure 2:**
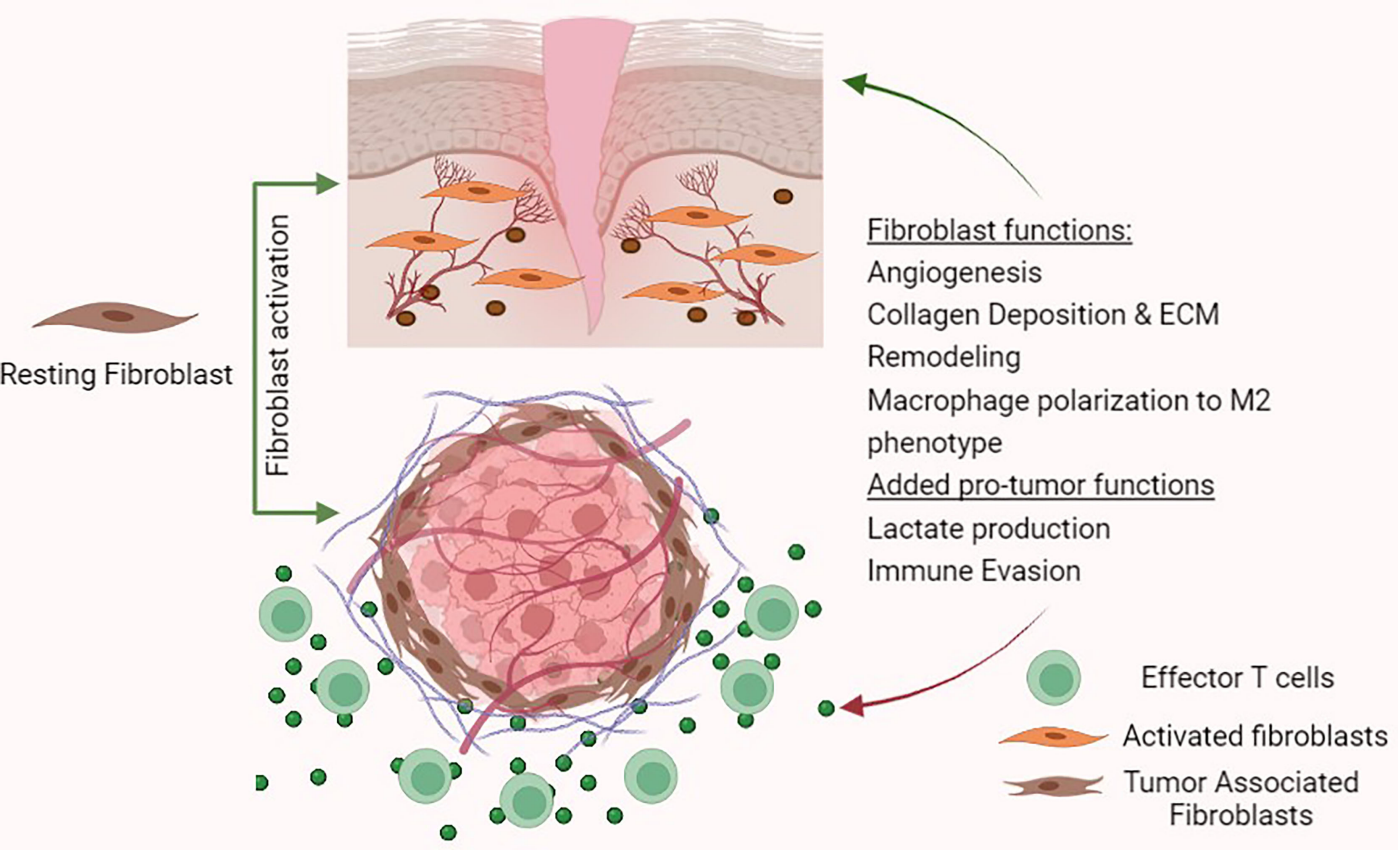
Fibroblast functions in wound healing and tumour- promoting functions in the tumour micro-environment.

TAFs are defined as fibroblast cells that surround both primary and metastatic cancers and represent one of the most abundant heterogeneous cell population in the tumour stroma. In general, fibroblasts as a cell type are difficult to identify since they lack specific and unique markers. Hence, identification of fibroblasts relies primarily on morphology and tissue location (9). Under resting conditions, fibroblasts appear as spindle shaped cells and are normally located in the interstitial space (7). Understanding the roles of fibroblasts in normal tissues can provide crucial insight into how cancers may hijack their functions for their own proliferation (**Figure 2**). Fibroblasts are otherwise quiescent cells that demonstrate functional phenotypes only after activation. Broadly, fibroblasts are key producers and modulators of ECM, regulators of tissue interstitial pressure, and are intimately involved in wound healing (7, 10). Underlying most fibroblast functions is the production of collagens and other ECM proteins along with metalloproteinases (MMPs) and tissue inhibitors of metalloproteinases (TIMPs) which together modulate ECM degradation (10). In wound healing, activated fibroblasts become stellate shaped and can gain pro-angiogenic functions to promote a pro-M2 secretome by producing factors such as VEGFA, TGF-β, IL-6 and CXCL-10. Tumours can capitalise on the release of these cytokines and growth factors to survive and grow (7).

Under the influence of the TME or in response to tissue injury caused by the growing tumour, fibroblasts can become activated to a distinct phenotype, and are termed tumour associated fibroblasts (TAFs) (also known as cancer-associated fibroblasts or CAFs) (9, 11, 12). TAFs can arise from quiescent tissue resident fibroblasts and from the activation of myofibroblasts. Alternatively, other cell types such as bone marrow derived mesenchymal stem cells (BM MSCs), pericytes, adipose, endothelial and epithelial cells can also differentiate into TAFs (9, 11, 13). Accurate tracing of TAF origin is important for differentiating between TAF subsets, which may have distinct phenotypes, and consequently allow specific targeting of pro-tumourigenic subsets.

### 2.2 Heterogeneity in TAF Populations in Cancer

The heterogeneity observed even in normal fibroblast populations gives us a fair idea of the complex fibroblast subtypes involved in cancer. Recent advances in single cell technologies have led to greater understanding of the heterogeneity in fibroblast subpopulations in the dermis (14, 15) and also in TAF populations along with the diversity of markers which are expressed by these subsets. Heterogeneity in TAF populations was also elucidated by the scRNA-seq of 52698 stromal cells isolated from lung tumours (with matching non-malignant controls). The study found five distinct TAF populations, each identifiable by its distinct set of associated collagen and ECM proteins (16). In other studies myofibroblasts and TAFs were identified to be present in tumour samples from head and neck squamous cancer patients through scRNA seq (17). Similar observations were made in colorectal cancer patients where normal fibroblasts, myofibroblasts and TAFs were identified (18). Other studies in cancer populations such as breast cancer have also noted the presence of multiple TAF subgroups, one of which had immune-regulatory function (14, 19). Largely, these studies explain the vast heterogeneity in TAF populations in different subsets of cancer and the need to identify specific markers for targeting TAF-related functions.

However, a common theme emerges from the wealth of information brought forward by single cell technologies, and based on these studies, TAFs can be broadly classified into two key subpopulations: a matrix-producing and contractile phenotype termed myTAFs and an inflammatory phenotype termed iTAFs. For a long time, myTAFs with an α-SMA positive signature were considered to be the only TAF population and these played a predominant role in the expression of ECM components, myofibroblast markers and cell contraction. With the advancement in single cell RNA sequencing technologies, inflammatory TAFs or iTAFs were identified as another distinct population characterized by an inflammatory secretome (20). TAFs have been predominantly implicated to be pro-tumour. However, some contradictory studies have also highlighted anti-tumour roles of TAFs. For example, myTAFs expressing α-SMA, have been observed to have anti-tumour properties in the TME in some studies (9, 21).

### 2.3 Tumour-Associated Fibroblasts: Activation in the Tumour Microenvironment

Understanding the multiple pathways that could lead to activation of the TAF phenotype is crucial to developing strategies that can prevent or even reverse this activation. Of the many factors released into the TME by cancer cells, TGF-β is one of the most well-known inducers of TAF activation, leading to the αSMA^+^ myofibroblasts (myTAFs) which have a pro-invasive phenotype (22). These activated TAFs engage in TGF-β and SDF-1 autocrine signalling loops that can amplify and maintain their activated phenotype (23). Additionally, TGF-β and SDF-1 produced by TAFs can subsequently lead to immune suppression (24) and angiogenesis (25) respectively. Although these changes may be a result of transcription by the downstream SMAD proteins, evidence suggests that reactive oxygen species (ROS) could play a prominent role in this pathway (26, 27). Importantly, TGF-β can increase the production of mitochondrial ROS, which in turn activates latent TGF-β and upregulates TGF-β gene expression, in a feed-forward loop (26). NADPH oxidases (NOXs) such as NOX4 are key producers of ROS downstream of TGF-β1 signalling (26). Other factors like chemotherapy can also indirectly contribute to the activation of fibroblasts and cause an increase in levels of TGF-β and IL-17A secretion (28) as a result of ROS production. In spite of its immune suppressive role, targeted inhibition of TGF-β may lack pathway specificity. Thus, inhibition of downstream players such as Nox4 could represent alternative targets for modulating TAF activation and polarisation. Other factors released in the TME like Platelet Derived Growth Factor (PDGF) signalling can also drive TAF activation (29, 30). Extracellular matrix (ECM) stiffness and organisation can also induce a pro-invasive phenotype *via* focal adhesion kinase (FAK) signalling (31). Activation of NF-κB, by IL-1β (32) or tumour derived exosomes (33), can also lead to a pro-inflammatory TAF phenotype in the TME. Overall, the different factors that could contribute to TAF activation suggest that differences in the resulting TAF populations may not be solely dependent on the cellular origin of the TAF subset, but also result from the combination of activating signals it receives in the TME. This could explain some of the phenotypic heterogeneity of TAFs seen within the TME. Recently, single cell profiling of stromal cells has revealed largely heterogenous populations of TAFs in pan cancer studies and along with specific TAF populations in conditions such as breast cancer, pancreatic ductal carcinoma and cholangiocarcinoma, that could possibly promote tumour cell growth through cellular crosstalk (5). Targeting TAF activation should thus take into consideration the dominant phenotype in the specific TME as well as alternative pathways to activation.

### 2.4 Tumour-Associated Fibroblast: Pro-Tumour Functions

#### 2.4.1 Extra-Cellular Matrix Remodelling

The ECM is composed of various collagens, proteoglycans and glycoproteins (34) and undergoes dynamic remodelling which is essential for development, wound healing and normal organ homeostasis. The ECM can change in composition and organisation to regulate both biochemical and biomechanical pathways (35). Life-threatening pathological conditions arise when ECM remodeling becomes excessive or uncontrolled (36). Cells can interact with the ECM *via* surface receptors, such as integrins, which affect cell adhesion and signalling (37) and through mechanical cues which can direct cell fate (38). The ECM can be divided into two forms: the basement membrane, which is a sheet-like structure surrounding cells and tissues that separates them from one another, and the interstitial matrix. Under conditions of wound healing, fibroblasts coordinate homeostatic ECM production and wound contraction (39). After completion of the repair process, they either undergo apoptosis or return to their quiescent state. However, this protective physiological process is dysregulated in cancer where TAFs retain their activated state and produce excessive amounts of collagen along with the collagen crosslinking enzymes like lysyl oxidase (LOX) and lysyl oxidase-like 1 (LOXL1), which increase ECM stiffness and direct tumour progression. On the other hand, upregulation of ECM degrading enzymes like MMPs can create spaces for migration (40) and release ECM bound factors such as TGF-β, fibroblast growth factor 2 (FGF-2) and vascular endothelial growth factor A (VEGFA). These factors can further increase angiogenesis and promote fibroblast activation along with ECM deposition in the TME (41). Additionally, the protein fibronectin is assembled into fibrils by TAFs, which allows directional migration of cancer cells (42), that is supportive of the pro-tumourigenic role of TAFs by modulating ECM remodelling.

#### 2.4.2 Angiogenesis

The production of the ECM is closely linked to the process of angiogenesis. Angiogenesis is the formation of new capillaries and blood vessels that are required to provide oxygen and nutrients to cells. Tumours cannot grow or metastasize without access to new blood vessels (43) and often even utilize abnormal vasculature to evade immune attack. The tumour vasculature consists of immature vessels that are hyper-permeable in nature. Abnormal tumour vasculature can result in decreased perfusion, hypoxia and can even promote glycolysis resulting in an acidic environment. This can potentially deter immune cell function. Recent evidence from RNA sequencing studies from 11,069 patients, have revealed that low angiogenic immune tumour types are more responsive to immune checkpoint blockade (44, 45). The balance of pro-angiogenic versus anti-angiogenic factors is crucial in characterizing the ‘angiogenic switch’, which can be triggered by hypoxia, mechanical stress, inflammatory cells or by stromal derived factors. In the normal wound healing process, fibroblasts promote angiogenesis to replace damaged vessels and restore nutrient supply to regenerating tissue. This is accomplished by VEGF production, which can be enhanced by the release of hypoxia-inducible factor-1alpha (HIF-1α), along with FGF-2 and TNFα (46). Other proteins such as MYCT1, expressed almost exclusively in tumour associated endothelial cells, have been identified to be regulators of angiogenesis. Targeting MYCT1 has been demonstrated to be synergistic with checkpoint blockade in inhibiting tumour growth in pre-clinical models (47). Tumour cells can capitalise on this new vascularization to maintain and promote their own growth. For example, TAF derived IL-6 has been shown to be critical in promoting angiogenesis in colon cancer through increased production of VEGFA in other TAFs and fibroblasts. This effect could be abrogated by the addition of an anti-IL-6 receptor antibody (48). In another study, it was observed that endothelial progenitor cells are recruited *via* TAF mediated SDF-1/CXCL12 release and signalling that can also lead to angiogenesis in growing tumours (49). Other cytokines such as CXCL8 (IL-8), a known pro-angiogenic factor, along with CCL2 have been implicated in increasing tumour angiogenesis in a mouse model of PDAC (50) These pre-clinical studies have therefore aided in establishing a link between cancer cells and their ability to direct TAFs to produce a variety of factors that support tumour growth *via* angiogenesis. However, in-spite of this growing body of evidence, the exact molecular mechanisms by which fibroblasts educate vascular cells in the TME remain to be completely elucidated.

#### 2.4.3 Metabolic Pathway Remodelling

A key hallmark of cancer cells is their ability to reprogram metabolism of glucose to produce lactic acid (“Warburg metabolism”) instead of oxidative phosphorylation. In the nutrient starved TME, cells need to adapt their metabolic pathways to survive and function efficiently. Some cells have the capacity to proliferate faster by having access to energy at a much higher rate through glycolysis. Cancer cells have long been thought to utilize glycolysis for energy supply, particularly in the (hypoxic) interior of tumours where oxygen cannot be supplied due to interrupted blood flow (51). More recently, certain types of immune cells under infectious stress have also been reported to similarly undergo glycolysis, converting glucose to lactic acid (52–54). This phenomenon is believed to provide uncertain advantages to cancer cell survival and influence functions of surrounding cells in the TME. Lactate is considered a key mediator in the metabolic crosstalk between cancer cells and the TME. For example, tumour-derived lactate contributes to tumour-associated macrophage (TAM) polarization towards the pro-tumour/anti-inflammatory phenotype (M2 type) (55). Angiogenesis can also be stimulated by increased extracellular lactate which can stabilise HIF-1α and increase NF-kB as well VEGF production (56). Cancer cells can induce myofibroblasts to perform Warburg metabolism for further generation of energy. Here, cancer cells induce Caveolin-1 deficient TAFs to conduct aerobic glycolysis and produce metabolites like lactate and pyruvate that cancer cells could then take up and process to derive energy for proliferation (57). This switch to aerobic glycolysis in fibroblasts can be induced by TGF-β1 or PDGF and is reflected in a decrease in the levels of isocitrate dehydrogenase 3α (IDH3α) (58). In an alternative pathway, cancer cells also secrete TGF-β1 to induce p38 MAPK signalling in TAFs, which can in turn release factors that promote the metabolism of glycogen to glucose in cancer cells, representing another source of ATP (59).

At a cellular level, an immune response requires substantial energy, necessitating metabolic changes to rapidly meet an urgent need for greater ATP production (60). Besides production of ATP for proliferation, the Warburg effect can also produce intermediate glucose metabolites that act as signalling molecules in regulating expression and activity of genes and proteins involved metabolic pathways and immunity (52, 54, 61, 62). We now know that immune and stromal cells including TAFs also share this functional phenotype. TAFs release pro-inflammatory cytokines including pro-type II cytokines such as thymic stromal lymphopoietin (TSLP) under the influence of unknown stimuli. TSLP-activated resident dendritic cells migrate to draining lymph nodes where they prime naïve T cells into CD4+ Th2 cells. Further, Th2 cells are recruited to the TME by chemo-attractants such as TARC and MDC that are released by activated DCs and tumour cells. Th2 cells also release cytokines IL-5 and IL-13 that foster fibrosis by increasing extracellular matrix deposition and development of M2-like TAMs. However, the specific triggers and receptors, and the signalling mechanisms, that effect metabolism and the immune response in TAFs and the TME are still obscure.

#### 2.4.4 Innate and Adaptive Immune Modulation

Apart from supporting cancer growth, angiogenesis, tumour metabolism and metastasis, recent evidence suggests that TAFs can also cripple the immune response to cancer and promote tumour immune escape by creating an immunosuppressive TME. For example, two TAF derived cytokines that have been implicated in multiple pathways of immune suppression are TGF-β and SDF-1/CXCL12. TAFs can therefore modulate the function of various immune cells in the TME, which have been discussed in the following sections.

##### 2.4.4.1 DC Activation

Among other antigen presenting cells, conventional dendritic cells (cDCs) are vital in initiating a T-cell response in cancer and represent the start of the cancer immunity cycle. DCs can endocytose dead cells and debris within the TME. Specifically, a small subset of cDC1s are required to cross-present antigens to CD8+ T cells (63). TAFs are major producers of growth factors like VEGF, which are known to inhibit the maturation of DCs (64). The relevance and mechanisms of this suppression in the TME are not completely understood. However anti-VEGF therapy has been shown to improve DC function and number (65, 66). Tumour metabolism can also influence DC differentiation, with lactate promoting tolerogenic, IL-10 producing DCs (67).

##### 2.4.4.2 DC Migration to Draining Lymph Node and Priming of T-Cells

TGF-β functions as a key inhibitor of DC migration and their ability to prime T cells. As discussed earlier, TAFs are major producers of TGF-β in many tumour types and also contribute significantly to the release of latent ECM bound TGF-β (24). TGF-β was shown to immobilise DCs in skin tumours, hence preventing their migration to lymph nodes for antigen presentation (68). TGF-β can also stimulate the downregulation of MHC class II expression and that of co-stimulatory molecules like CD40, CD80 and CD86. This results in immature DCs ultimately leading to the generation of immunosuppressive Tregs and tolerogenic T cells (69). Such T cell suppressive DCs are also characterized by the expression of the tryptophan degrading enzyme indoleamine 2,3-dioxygenase (IDO) (70). In TME, TAF derived IL-6 can stimulate the production of IDO in a STAT3 dependent manner and thereby contribute in decreased T cell immune response towards tumours (71).

##### 2.4.4.3 T-Cell Infiltration

T-cell infiltration into the tumour is an essential step that precludes T cell mediated killing of tumour cells, and thus, also represents a mode of resistance or lack of response to immunotherapies that aim to enhance T-cell activity. A subset of TAFs expressing fibroblast associated protein (FAP) have been documented to cause T-cell exclusion from cancer cell nests in multiple cancer models (72). Studies indicate that FAP+ TAFs produce CXCL12/SDF-1 that may be responsible for coating cancer cells and causing T-cell exclusion. Further evidence for the role of CXCL12 in T-cell infiltration came from other preclinical studies, where inhibition of CXCR4 (the receptor for CXCL12) enhanced the response to checkpoint inhibition in a pancreatic cancer model (73). However, it is important to note that in spite of these discoveries, the complete mechanism of T-cell exclusion propagated by TAF-mediated CXCL12/CXCR4 axis remains unclear.

##### 2.4.4.4 T-Cell Mediated Killing of Tumour Cells

TAFs can restrict cytotoxic T-cell mediated cancer cell killing by direct or indirect upregulation of checkpoint inhibition molecules in the TME. TAFs themselves exhibit immune checkpoint ligands. However, the impact this has on T-cell function is not clear (74). TAFs can also boost PD-L1 expression on other cells in the TME. For example, TAF derived CXCL5 was shown to upregulate PD-L1 expression on cancer cells in murine melanoma and colorectal cancer models (75). In animal models of hepatocellular carcinoma, TAFs were shown to induce PD-L1 expression in neutrophils through a IL-6 STAT signalling pathway (76). In other murine tumour models, TAFs were also identified to be the main source of CD73 which is another key immune checkpoint molecule (77). Collagen in the tumour ECM which is primarily secreted by TAFs, may also lead to CD8+ T-cell exhaustion and cause resistance to anti-PD-L1 therapy. In models of lung tumours, expression of the receptor LAIR1 on CD8+ T-cells caused by CD18 interaction with collagen was shown to prompt T-cell exhaustion through SHP-1 (78). Interestingly, studies have suggested that TAFs may protect tumours from T-cell responses by direct elimination of tumour specific CD8+ T-cells. These studies revealed an enhanced ability of TAFs to cross-present antigen from exogenous sources by complexing with MHC-I, which lead to T-cell death *via* interaction with immune checkpoints FAS and PD-1 in an antigen specific manner. On the contrary, normal fibroblasts did not exhibit this protection to tumours. Furthermore, non-deleted T-cells also showed increased expression of LAG3, a marker for exhaustion (79).

##### 2.4.4.5 Recruitment of Immunosuppressive Cells

TAFs can also influence the cancer immunity cycle indirectly by modulating recruitment and differentiation of immunosuppressive cell types in the TME. Macrophages recruited to the TME from circulating monocytes are called TAMs. These cells are particularly abundant, and in general promote tumour growth (80). In a rodent model of breast cancer, TAFs were observed to promote monocyte migration into the TME by secreting pro-inflammatory cytokines such as MCP-1 and CXCL12/SDF-1. Increased expression of CXCL12 chemokine receptor, CXCR4, was seen to be induced on monocytes by TGF-β (81). Unlike normal fibroblasts, TAFs were shown to coerce these monocytes to a more M2-like immunosuppressive phenotype, showing increased expression of markers associated with M2 macrophages such as CD163 and CD206, along with increased expression of PD-1 (82). A similar phenomenon of promoting M2 differentiation in macrophages was observed in other models of prostate cancer, PDAC, colorectal cancer and oral squamous cell carcinoma (83–86). Myeloid derived suppressor cells (MDSCs) are another immunosuppressive and pro-tumour immune cell population present in the TME that can aid in angiogenesis, metastasis and resistance to cancer immunotherapies (65). TAFs have been identified as recruiters of MDSCs in multiple rodent models of cancer. TAF derived SDF-1 was again implicated in the recruitment of MDSCs in murine breast and hepatocellular carcinoma models (87, 88). In a rodent model of lung squamous cell cancer, TAFs were found to recruit MDSCs *via* CCL2 (89). Most interestingly, contrary to TGF-β pro-tumour role in the TME, depletion of TGF-β resulted in increased MDSC infiltration in multiple studies (24).

Another population of adaptive immune cells that TAFs influence is that of the Foxp3+ regulatory T cell (Treg). Treg cells guard against autoimmunity and are well known to suppress anti-tumour responses. Infiltration of Treg cells into the TME is correlated with a worse prognosis in most cancers (90). In various cancers such as breast cancer, high grade serous ovarian cancer, and murine ovarian and pancreatic cancer models (91), a subset of TAFs were shown to associate with the recruitment and differentiation of Tregs in the TME, again *via* the secretion of SDF-1 (CXCL12) and its interaction with its receptor CXCR4. This effect was also seen in lung cancer models (92), however the study did not identify the exact mechanisms involved. Another cytokine that has been shown to induce upregulation of FOXP3 on CD4+ T cells to promote their differentiation into Tregs is TGF-β. This upregulation of FOXP3 on CD4+ T cells abrogated by TGF-β inhibition or by deletion of downstream transcription factors (24).

Stemming from the diverse roles that TAFs play in cancer survival and growth as well as immunosuppression, targeting TAFs for slowing disease progression in cancer could be potentially beneficial and enhance the effect immuno/chemo-therapy. In the following section, we describe current efforts and approaches targeting TAFs to sensitise the immunosuppressive TME and prevent tumour progression, chemoresistance and metastasis.

## 3 Towards Targeting TAFs in the Immunosuppressive Microenvironment

**Figure 3:**
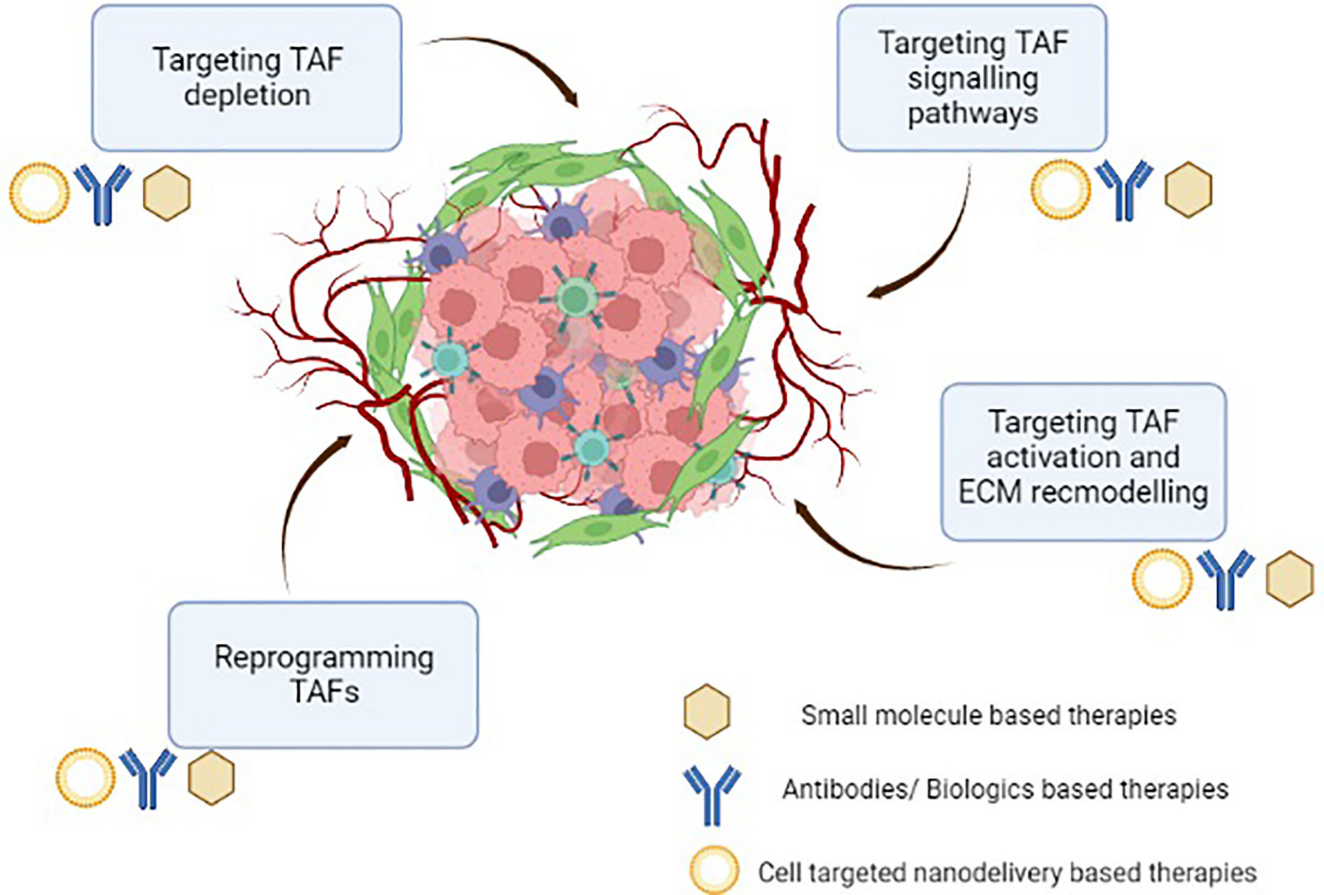
Therapeutic intervention targeting tumour-associated fibroblasts in the immunosuppressive tumour microenvironment.

TAFs regulate an array of functions in the TME. TAFs secrete various cytokines, chemokines, and growth factors and play an essential role in promoting tumourigenesis, angiogenesis and chemo resistance, etc. Due to their phenotypic and functional heterogeneity, they have essential and sometimes contradictory roles in both eradication and progression of tumours (21). Although no ubiquitous marker exists for studying the pathology of these fibroblasts, certain TAF-specific proteins such as alpha smooth muscle actin (α-SMA), fibroblast activation protein (FAP), fibroblast specific protein -1 (FSP-1), podoplannin (PDPN), and vimentin, insulin-like growth factor-binding protein-7 (IGFBP7) etc. have been utilized to identify these cells. These markers also provide an opportunity to specifically target various types of tumours by modulating TAF mediated cancer progression. Lately, the pro-tumourigenic role of TAFs has garnered a lot of attention, making them a potential therapeutic target for TME modulating cancer interventions (21). Additionally, TAFs are present in various cancer types and unlike tumour cells, they are genetically stable, thus ensuring that they do not acquire resistance to therapies. A number of promising therapies (small molecules, peptides, antibodies and nano-delivery systems) targeting TAFs for potentiating innate immune responses in the immunosuppressive TME have been developed (**Figure 3**) and are briefly presented in the following sections. We have categorized the representative therapeutic strategies, which are in either preclinical or clinical development stages into- (a) targeting cell surface markers leading to TAF depletion, (b) targeting TAF activation, signaling pathways and ECM remodeling, and (c) reprogramming strategies to de-differentiate and re-educate activated TAFs to normalized fibroblasts (**Figure 3**).

### 3.1 Targeting TAFs *via* Depletion

**Figure 4:**
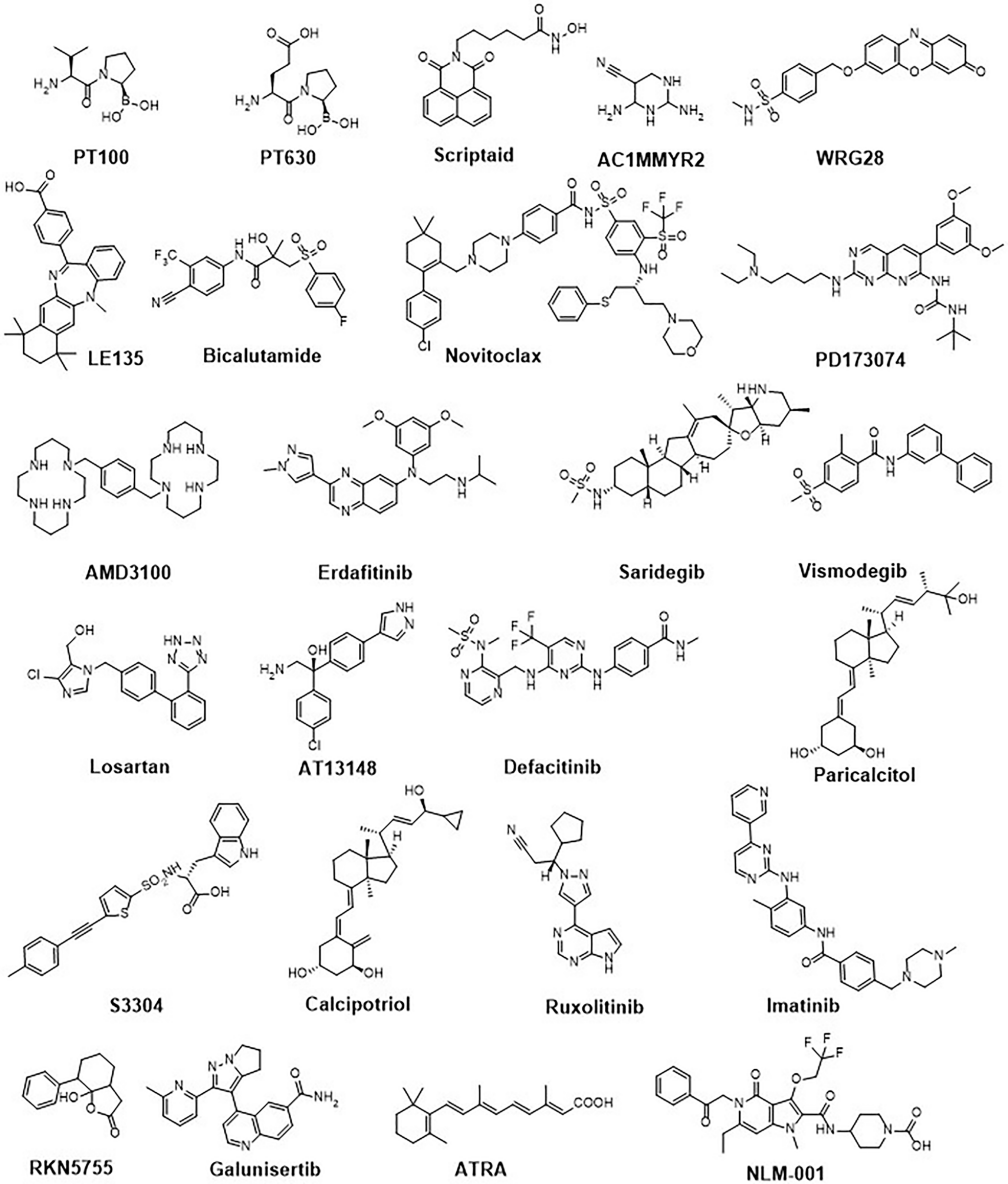
Chemical structures of a representative panel of small molecule drugs targeting tumour-associated fibroblasts.

Given the diverse role of TAFs in cancer progression, one probable way to target TAF function is to simply deplete their population, which can potentially alleviate TAF-mediated immunosuppression in the TME (93). Various strategies to target TAF depletion have been evaluated in preclinical models and clinical studies (**Figures 3**, **4**; **Table 1**). From these studies, targeting TAF population depletion through cell surface markers has emerged as the most promising approach.

**Table 1:** A representative panel of pharmacological interventions targeting tumour-associated fibroblasts.

No.	Therapies	Categories	TAF targeting strategy	Target	Targeting strategy/Mechanism	Disease	Development phase	References
1	PT100 in combination with Oxaliplatin	Small molecule	via Depletion	FAP	Inhibit TAF and reduce chemo-resistance	Colon Cancer	Discovery/ Preclinial	(94)
2	PT630	Small molecule	via Depletion	FAP	Inhibits tumour growth and stromagenesis	Lung and Colon Cancer	Discovery/Preclinial	(95)
3	FAP cDNA	DNA Vaccine	via Depletion	FAP	Inhibits tumour growth and pulmonary metastases	Colon Cancer	Preclinical	(96)
4	Sibrotuzumab	Anti-FAP mAb	via Depletion	FAP	Inhibit tumour growth	Advanced colorectal cancer	Clinical	(97)
5	FAP associated Chimeric antigen receptor (CAR) T cells	CAR T cell therapy	via Depletion	FAP	Inhibit tumour growth	Desmoplastic human lung cancer	Preclinical	(98)
6	Polymeric micelles + Vismodegib and Irinotecan	Delivery system	via Depletion	Hedgehog pathway	Inhibit the suppression of Glioma-associated protein-1 (GLI-1)	PDAC	Preclinical	(99)
7	Polymeric micelles + Cyclopamine and Paclitaxel	Delivery system	via Depletion	SMA/FAP	Enhanced angiogenesis and reduced hypoxia without depleting the collagenous matrix	PDAC	Preclinical	(100)
8	Nano-photoimmunotherapy (Ferritin + anti-FAP scFv + ZnF16PC)	Delivery system	via Depletion	FAP	Enhanced T-cell infiltration, followed by tumour suppression	Breast Cancer	Preclinical	(101)
9	PNP-D-mAb based micelles (with FAP mAb and CPP + Dox)	Delivery system	via Depletion	FAP	Specific tumour targeting and enhanced penetration capacity	Melanoma	Preclinical	(102)
10	Liposomes (FH-SSL-Nav) + Novitoclax and dox loaded transferrin lipsomes	Delivery system	via Depletion	Tenascin-C protein	Disrupting tumour and stroma interaction in TME and partly reversed the acquired drug resistance, leading to indirect tumour suppression	Hepatocellular carcinoma	Preclinical	(103)
11	Cellax NP (docetaxel and PEG conjugation)	Delivery system	via Depletion	SMA/SPARC	Decreased tumour IFP, increased perfusion and significantly suppressed lung metastases	Breast and Pancreatic cancer	Preclinical	(104)
12	Scriptaid	Small molecule	via Reprogramming	HDAC	Repress TGFB-mediated TAF differentiation *via* HDAC inhibition	Anti-cancer (disease not disclosed)	Discovery	(105)
13	AC1MMTYR2 (combined with taxol)	Small molecule	via Reprogramming	miR-21	Suppress tumour migration and invasion ability	Breast Cancer	Preclinical	(106)
14	WRG-28	Small molecule	via Reprogramming	Discoidin Domain Receptor 2 (DDR2)	Inhibit tumour invasion and migration	Breast Cancer	Preclinical	(107)
15	LE135 + Bicalutamide (in combination with cisplatin)	Small molecule	via Reprogramming	Retinoid acid receptor B and Androgen receptor	Inhibition of Chemo-resistance	Squamous cell carcinoma	Preclinical	(108)
16	Navitoclax	Small molecule	via Depletion	Bcl-2	TAF apoptosis, suppressing the expression of the desmoplastic extracellular matrix protein tenascin C, decrease tumour growth	Cholangiocarcin-oma	Preclinical	(109)
17	PD173074	Small molecule	via Depletion	FGFR	Decreased proliferation of fibroblasts and endothelial cells	Head and neck squamous cell carcinoma	Preclinical	(110)
18	AMD3100	Small molecule	via Depletion	CXCR4	Induce T-cell accumulation and depletes cancer cell	Pancreatic ductal adenocarcinoma (PDAC)	Preclinical	(73)
19	Erdafitinib	Small molecule	Prevents TAF activation	FGFR	Inhibition of tumour cell proliferation and tumour cell death	Advanced Urothelial cancer	Approved	(111)
20	Saridegib (in combination with Gemcitabine)	Small molecule	Prevents TAF activation	Hedgehog pathway	Reduce tumour incidence, slower growth and spontaneous tumour regression	Chondrosarcoma	Clinical trials ongoing	(112)
21	Vismodegib	Small molecule	Prevents TAF activation	Hedgehog pathway	Tumour growth inhibition	PDAC	Clinical trials ongoing	(113)
22	Losartan	Small molecule	Prevents TAF activation	Angiotensin receptor	Decrease level of ECM molecules, such as collagen and hyaluronan	Pancreatic Cancer	Phase II	(114)
23	AT13148	Small molecule	via Reprogramming	ROCK (Rho associated protein kinase)	Inhibition of ROCK based contractibility	PDAC	Phase I	(115)
24	Defactinib	Small molecule	via activation and then Reprogramming	FAK (Focal adhesion kinase)	Loss of FAK in TAFs, leads to reduced tumour growth and enhanced malignant glycolysis	Breast and Pancreatic cancer	ongoing clinical trials	(116)
25	Paricalcitol	Small molecule	via Reprogramming	Vitamin D receptor	Stellate cell normalization	Metastatic pancreatic cancer	Phase II	(117)
26	ATRA (All trans retinoic acid) in combination with Gemcitabine and nap Paclitaxel	Small molecule	via Reprogramming	Vitamin A metabolite	Stellate cell normalization	Pancreatic cancer	Phase Ib	(115)
27	Calcipotriol (in combination with Gemcitabine)	Small molecule	via Reprogramming	Vitamin D receptor	Reverse chemo-resistance would hinder tumour-stroma crosstalk and tumour growth	PDAC	Preclinical	(118)
28	Ruxolitinib (in combination with Capecitabine)	Small molecule	Prevents TAF activation *via* blocking JAK-STAT pathway	JAK 1 and JAK 2	Impair pro-tumourigenic activity of TAF and inhibits tumour growth	Metastatic pancreatic cancer	Phase II	(119, 120)
29	RKN5755	Small molecule	Prevents TAF activation	β-arrestin 1	Inhibit fibroblast activation by binding to β-arrestin 1	Breast cancer	Discovery	(121)
30	S3304	Small molecule	Prevents TAF activation/target TAF secretome	MMP (matrix metalloproteases)	Inhibition of tumour angiogenesis and metastasis	Advanced solid and refractory tumours	Phase II	(122)
31	Imatinib	Small molecule	Prevents signaling and activation	PDGFR (platelet derived growth factor)	Impaired tumour angiogenesis and cancer cell proliferation	Cervical cancer	Preclinical	(30)
32	Galunisertib	Small molecule	Prevents TAF activation	TGF β	Immunosuppression	Hepatocellular carcinoma	Phase II	(123)
33	NLM-001/TAK-441	Small molecule	Prevents TAF activation	Hedgehog pathway	Inhibit tumour growth	Pancreatic cancer and Medulloblastoma	Phase I	(124, 125)
34	131I-m81C6	Monoclonal Antibody	ECM remodeling	Tenascin-C protein	Inhibition of tumour metastasis	Recurrent malignant Glioma	Phase II	(126, 127)
35	Bevacizumab (in combination with Cisplatin	Monoclonal Antibody	Prevents TAF activation	VEGF	Impaired tumour angiogenesis/Immunotherapy	Pleural Mesothelioma	Phase III	(128, 129)
36	FG3019 (in combination with Gemcitabine)	Monoclonal Antibody	via Depletion	CTGF (connective tissue growth factor)	Immunotherapy/Enhance Chemo-resistance	PDAC	Preclinical	(130)
37	Simtuzumab	Monoclonal Antibody	ECM remodeling	LOXL2	Inhibit tumour growth	PDAC	Discontinued	(131)
38	FAP5-DM1	Antibody drug conjugate	via Depletion	FAP	Inhibition of tumour growth	lung, pancreas and head and neck cancers	Preclinical	(132)
39	αFAP-PE38	Immunotoxin	via Depletion	FAP	Inhibit tumour growth	Metastatic breast cancer	Preclinical	(133)
40	FAP peptide + Thapsigargin	Prodrug	via Depletion	FAP	Inhibit tumour	human cancer cells	Discovery/Preclinial	(134)
41	DC-shA20-FAP-TRP2	Vaccine	via Depletion	FAP	Improved tumour CD8+ T-cell infiltration and reduce antitumour activity	Melanoma	Preclinical	(135)
42	Simlukafusp alfa (RO6874281) (in combination with Pembroluzimab)	Antibody	via FAP inhibition	FAP	Mediate retention and accumulation in malignant lesions	Metastatic melanoma	Phase I	(136)
43	Bispecific liposomes (targeting FAP and HER2) + Trastuzumab	Delivery system	via FAP receptor targeting	FAP	Antibody therapy, addressed tumour plasticity, reduced antibody resistance	Melanoma	Preclinical	(137)
44	FAP cleavable peptide + Doxorubicin (CAP-NP)	Delivery system	via Depletion	FAP	Chemotherapy	Prostate cancer	Preclinical	(138)
45	HA@DSP-pep-DSP (Dox- loaded poly (amidoamine) nanoparticles	Delivery system	via Depletion	FAP	Chemotherapy	Prostate cancer	Preclinical	(139)
46	Dox/Pl-rGO + FAP cleavable peptide	Delivery system	via Depletion	FAP	Chemotherapy and cytotoxic peptide therapy	Colon cancer	Discovery	(140)
47	LPD liposomes (CXCL12 trap plasmid + PD-L1 trap plasmid) in combination with IL-1)loaded LPD nanoparticle	Delivery system	via Reprogramming	Sigma	Gene therapy and Immunotherapy. Inhibit triple negative breast cancer growth, prime the immune system	Breast cancer	Preclinical	(141)
48	FAP antibody+ CPP based nanoparticles loaded with siRNA and CXCL12 ligands	Delivery system	via Reprogramming and depletion of TAF	FAP	Inhibition of tumour cell invasion, migration and tumour angiogenesis	Prostate cancer	Preclinical	(142)
49	sTRAIL + LPD liposomes in combination with lipid coated cisplatin loaded nanoparticles	Delivery system	via Depletion	Sigma	Induce apoptosis in tumour cells	Bladder cancer	Preclinical	(143)
50	Nanocomplexes (dimeric CPP + anti-miRNA)	Delivery system	via Reprogramming	CPP	Inhibit the differentiation of PSC into TAFs	PDAC	Discovery	(144)

One such important cell surface marker in activated stromal fibroblasts is Fibroblast Associated Protein (FAP) that has garnered a lot of attention lately. FAP is a type-II membrane bound serine protease which has dipeptidyl peptidase (DPP) and collagenase-like activity and is important in remodelling the ECM. Various small molecules targeting the enzymatic activity of FAP have been explored in the past decade. Talabostat (also known as PT100), a boronic acid-based peptide inhibitor (from amino boronic dipeptide class) of FAP DPP activity, is the only FAP inhibitor to be tested in clinical studies. A combination of PT100 with Oxaliplatin reduced tumour growth and decreased recruitment of tumour associated macrophages in pre-clinical models of cancers (94). However, it is interesting to note that the compound did not elicit any clinically meaningful efficacy in Phase II clinical trials. Further, combination treatment of Talabostat (PT100) with docetaxel or cisplatin also did not demonstrate any effect on disease progression and survival in patients. Similar to PT-100, another amino boronic dipeptide inhibitor of FAP activity, PT-630 **(**GluBoroPro dipeptide), demonstrated retardation of tumour growth in preclinical animal models (95). Clinical efficacy of this compound is not known at present. Depletion of FAP+ TAF population using humanized antibodies targeting has also been evaluated in a number of studies. Pre-clinical efficacy studies were conducted by Kraman et al. (2010), where stromal cells expressing FAP were conditionally deleted. In animal models of lewis lung carcinoma and pancreatic ductal carcinoma, this selective ablation led to control of tumour growth (145). A FAP targeting strategy was also employed by Gottschalk et al. (2013) who developed a compound DC vaccine with three targets: silencing of the zinc finger A20 to improve DC activation, targeting of TAFs through FAP, and targeting of the tumour antigen tyrosine related protein 2 (TRP2). The authors observed T-cell responses towards FAP and TRP2, enhanced CD8+ T-cell infiltration and antigen spreading, leading to potent anti-tumour activity (135). Similar CD8+ T cell mediated effects were seen in another DC vaccine targeting FAP in a CT26 mouse colon cancer model (96). A few of these FAP targeting antibodies have also been taken up in clinical trials with varying success. One such example is Sibrotuzumab which is the humanized form of the murine anti-FAP mAb F19. Sibrotuzumab was evaluated in a Phase I clinical trial involving patients with metastatic colorectal cancer, but showed little or no clinical benefit (97). It is worth mentioning here about the FAP targeting antibody Simlukafusp alfa (RO6874281; developed by Roche) which is a novel monomeric bispecific IL-2v immunocytokine having picomolar range binding affinity to FAP. A key advantage of this concept is that apart from binding to IL-2R expressed on immune effector cells, binding to FAP allows for the retention of IL-2v in the tumour, which can further affect immune cells in the TME. A number of phase I trials of Simlukafusp alfa as a single agent or in combinations are currently underway (146) (ClinicalTrials.gov identifiers: NCT03875079, NCT03063762, NCT02627274, NCT03386721 & NCT03193190). Next, another approach to target FAP+ TAFs was utilized by Ostermann et al. (2008) where an antibody-maytanisoid conjugate FAP5-DM1 containing anti-FAP-mAb was covalently linked to a tubulin binding maytansinoid with anti-mitotic activity (DM1). Treatment with FAP5-DM1 lead to increased apoptosis of malignant epithelial cells and also induced long lasting tumour inhibition in mice xenograft models of lung, pancreas, head and neck cancers (132). Other studies targeted FAP+ TAFs using a FAP-targeted immunotoxin αFAP-PE38. In a mouse model of metastatic breast cancer (4T1), treatment with αFAP-PE38 led to significant reduction in the recruitment of tumour infiltrating immune cells and tumour growth as monotherapy and in combination with paclitaxel (133).

Alternatively, the adoptive transfer of FAP targeted CAR T-cells is also an exciting approach for targeting TAFs in TME. In a study by Wang et al. (2014) and Lo et al. (2015), the use of FAP targeted CAR T cells produced inhibition of cancer growth both by improving CD8+ T-cell function (147), and by reducing ECM proteins and vascular density without eliciting severe toxicity in their models. However, targeting FAP+ cells can be non-selective towards cancer due to the wide expression of FAP in different cells. It was further observed that FAP targeted CAR T-cells elicited lethal toxicity and cachexia due to reactions against bone marrow stromal cells, which also express FAP on the cell surface (148). In support of this, studies using bioluminescent imaging in mice revealed that FAP+ cells were found in most tissues and their depletion led to cachexia and anemia. In the face of such potential systemic toxicity concerns, nanoparticle-aided targeted delivery of FAP targeting therapies are currently being explored to minimize toxicity and improve tumour specificity (149). For example, Zhen et al. (2017) have described the use of ferritin (a protein nanocage) conjugated with a FAP targeted single chain variable fragment that can attach to FAP+ cells selectively in the TME. Further, localized photo-irradiation enabled killing of FAP+ TAFs in TME are being evaluated to provide efficacy without causing significant toxicity (101). In another study, PNP-D-mAb based nano-delivery system, was evaluated to selectively target TAF in the TME. In this system, a cell penetrating peptide (CPP) was synthesized with nine arginine residues and cholesterol was embedded in it to provide as a hydrophobic tail. These modifications resulted into the self-assembly of this peptide to form peptide nanoparticles (PNP) and doxorubicin was encapsulated into this construct to form PNP-D. Finally, these particles were modified to construct PNP-D-mAb (102) that demonstrated preclinical efficacy by targeting FAP in both *in vitro* and *in vivo* studies. In another study, bispecific targeted liposomes combining single chain antibody fragments specific for FAP along with either variable antibody fragments or Trastuzumab for targeting HER2 were developed. Treatment with these bispecific liposomes showed greater depletion of HER2 expressing tumour cells and aided in tumour specific distribution in pre-clinical models (137). Aside from directly targeting FAP receptors, use of a novel cleavable amphiphilic peptide (CAP) as a substrate for FAP has also been explored in preclinical studies. These self-assembling peptides can form nano-fiber like structures in aqueous solution and enable further loading of cytotoxic drugs, which can be delivered upon FAP mediated cleavage of the particle (138). On similar lines, Hou and colleagues (2019) designed a nanoparticle (HA@DSP-pep-DSP) based on the FAP cleavable peptide DATGPA, from which dox loaded poly (amidoamine) particles were released (mediated by glutathione) and accumulated in the tumour stroma (139).

Several other cell surface markers which can help define TAF subsets have also been evaluated for selectively targeting TAFs in the TME. Among small molecules, Navitoclax, an inhibitor of the programmed cell death regulator BCL-2 that functions as a pro-apoptotic factor, has been shown to induce cell death in activated fibroblasts. Navitoclax is currently under investigation as an anti-fibrotic and anti-cancer agent, with phase II and III clinical trials underway (150). Similarly, antibodies targeting other TAF specific cell surface receptors have also been explored. Su et al. (2018) described a subset of TAFs expressing the surface markers CD10 and GPR77, which was found to promote survival of cancer stem cells in breast cancers. In CD10^+^GPR77^+^ -TAFs, NF-κB signalling *via* p65 phosphorylation leads to IL-6 and IL-8 secretion, that aids in the development of chemoresistance. However, this effect could be reversed by blocking GPR77 with a neutralizing antibody (151). Another approach that has been evaluated is targeting connective tissue growth factor (CTGF) using monoclonal antibodies. The monoclonal antibody FG-3019 targeting CTGF was investigated in a murine model of PDAC. Treatment with FG-3019 in combination with gemcitabine, led to increased killing of cancer cells through the modulation of the TME and not solely due to the increase in delivery of chemotherapy to the tumour site (130). Other studies have evaluated the role of fibroblast growth factor (FGFR) in conditions such as head and neck squamous cell cancers (HNSCC). The FGFR inhibitor PD173074 restrained fibroblast and endothelial cell proliferation and led to suppressed growth of tumour cells both *in vitro* and in a murine xenograft model of HNSCC (110).

Multiple studies have employed nanoparticles for targeted delivery of drugs to the TME and the same has been extended to targeting TAFs. For example, TAF targeted micelles incorporating Vismodegib, an abnormal sonic hedgehog (SHH) signaling inhibitor and SN38 (active metabolite of an anticancer drug Irinotecan) encapsulated in a polymeric PEG_5k_-P (HEMASN 38)_x_ nanoparticle, have been studied (99). Other SHH inhibitors like cyclopamine were similarly co-delivered with Paclitaxel and this resulted in reduction αSMA^+^ (28%) and FAP-α^+^ (56%) TAF populations (152). Chen and colleagues reported the use of a novel TAF targeted nanovesicles (FH-SSL-NV) to completely deplete TAF and enhance the antitumour activity of Doxorubicin. The authors used the FH-peptide which targets tenascin C protein that is highly expressed in TAFs. This peptide was conjugated to prepare TAF targeted Navitoclax loaded liposomes (103). In addition, there are other receptors overexpressed on TAFs, for example SPARC which is an albumin binding protein, highly overexpressed in some desmoplastic tumours (prostate and breast cancers). Li and colleagues have utilised this approach of targeting SPARC by using Cellax. Cellax is a polymeric nanoparticle that is synthesised by conjugating docetaxel and PEG. Cellax was found to be more effective in depleting stroma in the TME as compared to docetaxel and nab-paclitaxel (104).

However, it is pertinent to re-emphasize that the heterogeneity in TAF populations and their specific context roles in tumour progression needs to be taken into consideration before translating TAF targeted therapies to the clinic. Indiscriminate TAF depletion may in fact lead to worse outcomes. This was observed in a murine model for pancreatic cancer, where depletion of αSMA^+^ myofibroblasts unexpectedly resulted in reduced animal survival. The resulting highly invasive tumours were characterized with higher levels of hypoxia, epithelial to mesenchymal transition (EMT) and cancer stem cell-like phenotype, along with increased recruitment of immunosuppressive Foxp3^+^ Tregs (93). Another study using a murine model of PDAC showed that SHH deficient mice with lower stromal content displayed more aggressive and proliferative tumours with increased vascularity (113). Therefore, enhanced specificity in targeting TAFs in the TME is warranted and other potential approaches to achieve this are discussed in the next two sections.

### 3.2 Targeting TAF Activation, Signaling and ECM Remodeling

An alternative to depleting TAF populations can be to neutralize their effects on the TME by hampering TAF activation, related signaling, or their ability to regulate the ECM. This approach was utilized by Shen et al. (2018) to inhibit TAF activation related signaling. The authors used engineered lipid-protamine-DNA (LPD) nanoparticles carrying trap genes for IL-10 and CXCL12 to alleviate T cell inhibition. In pre-clinical studies, this approach led to changes in ECM remodeling with decreased levels of collagen and α-SMA as well as fewer immunosuppressive cells in a murine PDAC model (73, 141). Inhibition of CXCL12 released from FAP expressing TAFs can be another potential method to alleviate immune suppression. For example, the pre-clinical efficacy of a clinically approved inhibitor of CXCL12 receptor CXCR4, AMD3100, has been evaluated in various animal models of cancer. However, due to its limited efficacy as monotherapy, a combination therapy regimen has been employed with anti-PD-L1 and anti-CTLA4 immunotherapy (73, 153), docetaxel chemotherapy (154), radiation therapy (155) and a siRNA based lipid nanoparticle system (156) in preclinical studies. AMD3100 was synergistic with these various combinations and suppressed tumour growth in these studies. Lang et al. developed a cell-penetrant peptide based nanoparticle which targets TAFs through FAP-α antibody and delivers siRNA targeting CXCL12 into the TME. In an animal model of prostate cancer, this nanoparticle decreased tumour cell invasion, migration and angiogenesis accompanied by a reduced potential for metastasis (142). In order to target matrix metalloproteinases (MMPs), a key mechanism by which fibroblasts orchestrate ECM deposition, Chiappori et al. (2007) conducted a phase I clinical trial of the MMP inhibitor S-3304. In this study, S-3304 was found to be safe and well tolerated (124). The detailed efficacy of S-3304 is yet to be evaluated.

Targeting intracellular signaling in TAFs to modulate their phenotype is another potential therapeutic approach to modulate the TME. Suvarna et al. (2018) have targeted fibroblast migration using RKN5755, a ligand for beta-arrestin1 to interfere with downstream cofilin signaling pathways and restrict the migratory phenotype of activated fibroblasts (121). In another study, platelet derived growth factor (PDGF) receptor signaling was inhibited using imatinib in a mouse model of cervical carcinogenesis. This inhibition led to the reduced expression of fibroblast growth factor 2 (FGF-2) and FGF-7 leading to suppression tumour growth (30). A number of other agents targeting TAFs are also under investigation in clinical trials. Losartan, an angiotensin II blocker, capable of inhibiting the TGFβ pathway in TAFs to prevent their activation was evaluated in the clinic. Losartan treatment led to reduced levels of collagen and hyaluronan, consequently causing hypoxia in the TME. A phase II clinical study combining losartan with chemotherapy and radiotherapy reported increased R0 resection rates (157). Another phase II clinical study with losartan in combination with immunotherapy is also underway (ClinicalTrials.gov Identifier: NCT03563248). Similarly, using Galunisertib, a TGF-β receptor type I inhibitor, in combination with sorafenib showed an acceptable safety profile and an improved overall survival in patients with advanced hepatocellular carcinoma enrolled in a phase II human clinical trial (123). Other compounds like Erdafitinib, which is a pan fibroblast growth factor receptor (FGFR) inhibitor, is already approved for use in patients with advanced urothelial cancer having specific FGFR mutations (111). Angiogenesis in cancer is critical for its survival, growth and also metastasis. This is one of the key functions mediated by TAFs that can be a potential therapeutic strategy. In a Phase III clinical trial aimed to evaluate a combination of bevacizumab (VEGF inhibitor) and cisplatin plus pemetrexed which is the standard of care regimen for advanced malignant pleural mesothelioma, an increase in the OS was observed. However, this combination therapy did lead to increased toxic adverse effects (128).

In summary, Targeting TAF derived cytokines and other upstream signaling modulators remains challenging in spite of the promise it holds as a potential therapeutic approach. Given the various different mechanisms by which TAFs can promote tumour progression— multiple cytokines, metabolites, exosomes, force-mediated contraction, and even direct contact with cancer cells and transcytosis (158)— the scope of effects meditated by these single inhibitors, even in combinations remain unclear and needs further investigation.

### 3.3 TAF Reprogramming and Normalization

Another approach to deter TAF function in the TME is to target fibroblasts which have already differentiated into a tumour promoting phenotype and to reprogram them to their native quiescent state. This reprogramming of TAFs has been attempted at the transcriptional level by several researchers. For example, Kim et al. identified the small molecule Scriptaid, an HDAC 1/3/8 inhibitor, as a factor that can reduce TGF-β-induced TAF differentiation. This in turn led to lower ECM secretion, cell invasiveness and stiffness in preclinical animal models (105). Use of DNA methyl transferase (DNMT) inhibitors along with JAK inhibition has also demonstrated preclinical efficacy in reverting TAFs to a wild-type phenotype by targeting the proinflammatory cytokine leukemia inhibitory factor (LIF) (159). Another study identified the methyltransferase nicotinamide N-methyltransferase (NNMT) enzyme as a central regulator of TAF activation in the TME. Here, treatment with an NNMT inhibitor alone decreased tumour burden in a mouse model of ovarian cancer (160). Chan et al. targeted two nuclear receptors (ligand responsive transcription factors), retinoid acid receptor β and androgen receptor, with their respective antagonists LE135 and bicalutamide in combination with cisplatin therapy to abrogate chemotherapy resistance in a mouse xenograft model of squamous cell carcinoma (SCC) (108). Ren et al. (2016) combined AC1MMTYR2, a small molecule inhibitor of miR-21, with taxol and were able to observe reduced tumour migration and invasion in rodent models of cancers. The authors also reported lower levels of FAP and α-SMA in a mouse model of breast cancer (106). In a mouse model of metastatic breast cancer, the small molecule WRG-28 was shown to inhibit the collagen receptor discoidin domain receptor 2 (DDR2) and block communication pathways between the tumour, stromal cells and the ECM. Following treatment with WRG-28, a reduction in tumour migration and invasiveness was also observed in these studies (107). Other reports involve inhibition of Rho-associated protein kinase, a small GTPase involved in cell contractility, that is upregulated in PDAC. Treatment with inhibitors of Rho-associated protein kinase, Fasudil and AT13148, resulted in reduced tumour growth and invasiveness in animal models of cancers (115). Paricalcitol, a vitamin D analog, has been shown to revert pancreatic stellate cells (PSCs) to a non-activated phenotype (161). Another vitamin D receptor ligand, Calcipotriol, yielded similar results (118). Since the deficiency of vitamin A has been observed in PDAC and it is known to play a role in PSC activation, ATRA treatment was initiated in a murine disease model and in human samples to reprogram PSCs to their quiescent state (162). Similarly, Han et al. (2018) also employed ATRA along with siRNA targeting heat shock protein 47 (HSP47), using a gold nanoparticle construct, to induce PSC quiescence in a PDAC xenograft model (163).

Aside from these, kinases involved in the complex signalling network in TAFs can also be potential targets for inhibition of TAF function. A multi-kinase receptor inhibitor Nintedanib was shown to downregulate the induction of collagens and α-SMA in TGF-β1 stimulated fibroblasts (164). Borriello et al. investigated the effect of inhibiting JAK2/STAT3 and MEK/ERK/1/2 using ruxolitinib and trametinib in a murine model of neuroblastoma to reverse the activation of a subset of pro-tumourigenic TAFs. They observed an increase in response to etoposide and overall survival (119). Ford et al. focused on inhibition of NOX4, a ROS-producing enzyme which is a downstream target of TGF-β1, and can regulate TAF phenotype. Interestingly, TGF-β inhibition could prevent myofibroblast differentiation, but not revert activated TAF phenotype to normal fibroblasts. On the contrary, NOX4 inhibition using Setanaxib (GKT137831) could revert TAFs to their normal phenotype. Treatment with Setanaxib also improved CD8+ T-cell infiltration which was initially limited to the periphery in TAF-rich tumours but increased to the center of tumours upon normalization. Consequently, tumours rich in TAFs were resensitized to anticancer vaccination and PD-1 checkpoint inhibition (165). Miao et al. made clever use of the off target uptake of anticancer nanoparticles by fibroblasts by creating and delivering plasmids encoding sTRAIL— a secretable form of TNF-related apoptosis-inducing ligand, which can induce apoptosis selectively in cancer cells. Thus, a majority of TAFs were reprogrammed to factories of sTRAIL secretion, which led potent tumour suppression in these preclinical studies. They also observed a normalization of residual TAFs to a quiescent state, which further inhibited tumour growth (166). Overall, multiple approaches have been utilized in preclinical and clinical studies to target the pro-tumourigenic role of TAFs including their effective depletion from the TME, inhibition of signaling pathways and their reprogramming to varying success. The heterogeneity of TAF population in the TME poses a big challenge to specifically target the pro-tumourigenic populations while not effectively deleting their anti-tumour properties. With the identification of TAF specific roles and markers along with the advent of newer targeting approaches, we anticipate that a greater success at therapy can potentially be achieved in the coming years.

## 4 Conclusions and Future Directions

Overall, fibroblasts are a dominant stromal cell type present in many tumours, which can be activated within the TME and ‘educated’ by cancer cells into overall tumour-promoting TAFs. Usually, quiescent fibroblasts become activated in tissue repair where they manipulate the ECM, regulate new vessel formation, and generate a skew towards an immunosuppressive, wound healing immune phenotype. Once the repair is complete, activated fibroblasts revert back to a normal phenotype. The continued activation of fibroblasts is thus characteristic of pathological responses such as organ remodelling and fibrosis, as well as tumours and cancers. Within the TME, cancer cells can program fibroblasts to pro-tumourigenic TAFs, which take on unique, heterogenous phenotypes, and are activated by different factors, including TGF-β. Further research is warranted to completely understand TAF differentiation and distinctions need to be made between the various subsets of TAFs that may influence tumour progression and metastasis in unique ways. The major hurdle for specifically targeting pro-tumourigenic TAF subsets is identifying reliable and specific markers that have largely remained elusive to date.

TAFs are capable of promoting tumour progression in many ways, which include ECM modulation, promoting angiogenesis, modulating the metabolic environment and creating an immunosuppressive TME. However, some mechanisms involved in these processes remain yet to be elucidated. For example, a more concrete understanding of how TAFs affect metabolism of tumour cells and other stromal cells, such as immune cells, is vital. Similarly, their impact on different stages of the cancer immunity cycle should be carefully examined to uncover opportunities for combination treatments. Despite the bulk of evidence that suggests that key TAF subsets support tumour progression, it is interesting that non-specific TAF depletion leads to pro-tumour responses, while re-educating TAFs and reversal to a normalized phenotype is protective. This warrants closer study of phenotypes and signalling mechanisms involved in the anti-tumour functions of certain TAF subsets. Various preclinical studies indicate that TAF content in common syngeneic murine models is lower than in human tumours. Examining TAF content and developing TAF rich preclinical tumour models that mimic human tumours may make them more accurate and reveal further details of TAF influence on tumour progression, metastasis and chemo-resistance. It is likely that characterizing and targeting the tumour stromal component may become essential in future cancer treatment, and reprogramming TAFs could unlock the potential of many existing therapies. Additionally, positive outcomes from the various ongoing clinical trials compiled in **Table 2** will ultimately help unlock the true clinical potential of targeting TAFs in regulating the TME in various human cancers.

**Table 2:** A representative panel of current clinical trials with either solid tumour microenvironment modifying or tumour-associated fibroblasts targeting agents in combination with FDA-approved immune-oncology therapies.

Target	Combination drug names		Modalities	Development Phase	NCT identifier No.	Indication
Fibroblast Activation Protein (FAP)-α	RO-7122290	Cibisatamab	Bispecific antibody targeting 4-1BB & FAP	Phase I/II	NCT04826003	Metastatic colorectal cancer
		Multiple immunotherapies			NCT03869190	Urothelial Carcinoma
	Talabostat	Pembrolizumab	Peptide inhibitor of FAP	Phase I/II	NCT04171219	Advanced Solid Cancers
		Pemetrexed			NCT00290017	Stage IIIB/IV Non-Small Cell Lung Cancer (NSCLC)
		Gemcitabine			NCT00116389	Stage IV Adenocarcinoma of the Pancreas
		Docetaxel			NCT00080080	Advanced Non-Small Cell Lung Cancer
		Temozolomide or Carboplatin			NCT00303940	Relapsed or Refractory Brain Tumours or Other Solid Tumours
		Doxetaxel			NCT00243204	Stage IIIB/IV Non-Small Cell Lung Cancer (NSCLC)
		Cisplatin			NCT00083252	Advanced Melanoma
		Rituximab			NCT00086203	Advanced Chronic Lymphocytic Leukemia (CLL)
	RO7300490	Atezolizumab	Fibroblast Activation Protein-α (FAP) Targeted CD40 Agonist	Phase I	NCT04857138	Advanced Solid Tumours
	NG-641	Nivolumab	Oncolytic adenoviral vector delivery system for targeting FAP	Phase I	NCT05043714	Metastatic or Advanced Epithelial Tumours
		Pembrolizumab			NCT04830592	Squamous Cell Carcinoma of the Head and Neck
	Simlukafusp Alfa (RO6874281)	Pembrolizumab	Immunocytokine containing an antibody against FAP	Discontinued	NCT03875079	Advanced Or Metastatic Melanoma
		Atezolizumab or Bevacizumab			NCT03063762	Unresectable Advanced and/or Metastatic Renal Cell Carcinoma (RCC)
		Trastuzumab or Cetuximab			NCT02627274	Solid Tumour; Breast Cancer; Cancer of Head and Neck
		Atezolizumab or Gemcitabine or Vinorelbine			NCT03386721	Advanced and/or Metastatic Solid Tumours
		Multiple immunotherapy			NCT03193190	Metastatic Pancreatic Ductal Adenocarcinoma
Fibroblast Growth Factor	Erdafitinib	Enfortumab Vedotin	Small molecule inhibitor	Launched	NCT04963153	Metastatic Bladder Cancer
		JNJ-63723283			NCT03547037	Advanced Solid Cancers (Japanese patients)
		Abiraterone Acetate or Enzalutamide			NCT03999515	Double Negative Prostate Cancer
		Combination with different chemotherapy regimens			NCT04172675	High Risk Non-Muscle-Invasive Bladder Cancer (NMIBC)
		Fulvestrant and Palbociclib			NCT03238196	ER+/HER2-/FGFR-amplified Metastatic Breast Cancer
		Vinflunine or Docetaxel or Pembrolizumab			NCT03390504	Advanced Urothelial Cancer and Selected Fibroblast Growth Factor Receptor (FGFR) Gene Aberrations
Hedgehog Signalling Pathway	Vismodegib (GDC-0449)	GSK2256098, Capivasertib & Abemaciclib	Small molecule inhibitor	Launched	NCT02523014	Progressive meningiomas
		ASN-002			NCT04416516	Basal Cell Carcinoma and Basal Cell Nevus Syndrome
	Saridegib (IPI-926)	Gemcitabine	Small molecule inhibitor	Phase III	NCT01130142	Metastatic Pancreatic Cancer
		FOLFIRINOX			NCT01383538	Advanced Pancreatic Adenocarcinoma
		Cetuximab			NCT01255800	Recurrent Head and Neck Cancer
Focal Adhesion Kinase (FAK)	Defactinib	Pembrolizumab	Small molecule inhibitor	Phase I/II	NCT04201145	Pleural Mesothelioma
		VS-6766			NCT04625270	Recurrent Low-Grade Serous Ovarian Cancer With and Without a KRAS Mutation
					NCT04720417	Metastatic Uveal Melanoma
					NCT04620330	Recurrent G12V or Other KRAS-Mutant Non-Small Cell Lung Cancer
		Paclitaxel			NCT01778803	Advanced Ovarian Cancer
		RO5126766			NCT03875820	NSCLC, Solid Tumour, Low Grade Serous Ovarian Cancer, Colorectal Cancer
		Pembrolizumab			NCT02758587	Advanced Solid Malignancies
					NCT03727880	Resectable Pancreatic Ductal Carcinoma
		Pembrolizumab and Gemcitabine			NCT02546531	Advanced Cancer
		Paclitaxel and Carboplatin			NCT03287271	Carboplatin resistant ovarian cancer

## Author Contributions

KS initiated and wrote the manuscript and was the major content provider. KS, SB, PG and AI contributed to determining the content of each section. KS, SB, PG and AI collected data and contributed to writing each section of the manuscript. All authors contributed to editing and have read and approved the final manuscript.

## Funding

This research was supported by Alembic Pharmaceuticals, India including salary support to SB, PG and AI. The funder was not involved in the study design, collection, analysis, interpretation of data, the writing of this article or the decision to submit it for publication.

## Conflict of Interest

Authors KS, SB, PG and AI were employed by the company Alembic Pharmaceuticals Ltd., India.

The remaining authors declare that the research was conducted in the absence of any commercial or financial relationships that could be construed as a potential conflict of interest.

## Publisher’s Note

All claims expressed in this article are solely those of the authors and do not necessarily represent those of their affiliated organizations, or those of the publisher, the editors and the reviewers. Any product that may be evaluated in this article, or claim that may be made by its manufacturer, is not guaranteed or endorsed by the publisher.
